# Performance of a RAD51-based functional HRD test on paraffin-embedded breast cancer tissue

**DOI:** 10.1007/s10549-023-07102-y

**Published:** 2023-09-19

**Authors:** Lise M. van Wijk, Sylvia Vermeulen, Natalja T. ter Haar, Claire J. H. Kramer, Diantha Terlouw, Harry Vrieling, Danielle Cohen, Maaike P. G. Vreeswijk

**Affiliations:** 1 Department of Human Genetics, Leiden University Medical Center, 2300 RC Leiden, The Netherlands; 2 Department of Pathology, Leiden University Medical Center, 2300 RC Leiden, The Netherlands

**Keywords:** Breast cancer, Homologous recombination deficiency, RAD51-FFPE test, RECAP test, *BRCA-*deficiency and biomarker

## Abstract

**Purpose:**

*BRCA-*deficient breast cancers (BC) are highly sensitive to platinum-based chemotherapy and PARP inhibitors due to their deficiency in the homologous recombination (HR) pathway. However, HR deficiency (HRD) extends beyond *BRCA*-associated BC, highlighting the need for a sensitive method to enrich for HRD tumors in an alternative way. A promising approach is the use of functional HRD tests which evaluate the HR capability of tumor cells by measuring RAD51 protein accumulation at DNA damage sites. This study aims to evaluate the performance of a functional RAD51-based HRD test for the identification of HRD BC.

**Methods:**

The functional HR status of 63 diagnostic formalin-fixed paraffin-embedded (FFPE) BC samples was determined by applying the RAD51-FFPE test. Samples were screened for the presence of (epi)genetic defects in HR and matching tumor samples were analyzed with the RECAP test, which requires ex vivo irradiated fresh tumor tissue on the premise that the HRD status as determined by the RECAP test faithfully represented the functional HR status.

**Results:**

The RAD51-FFPE test identified 23 (37%) of the tumors as HRD, including three tumors with pathogenic variants in *BRCA1*/*2*. The RAD51-FFPE test showed a sensitivity of 88% and a specificity of 76% in determining the HR-class as defined by the RECAP test.

**Conclusion:**

Given its high sensitivity and compatibility with FFPE samples, the RAD51-FFPE test holds great potential to enrich for HRD tumors, including those associated with *BRCA*-deficiency. This potential extends to situations where DNA-based testing may be challenging or not easily accessible in routine clinical practice. This is particularly important considering the potential implications for treatment decisions and patient stratification.

**Supplementary Information:**

The online version contains supplementary material available at 10.1007/s10549-023-07102-y.

## Introduction

Breast cancer (BC) accounts for 30% of newly diagnosed female cancers and is the second highest leading cause of cancer death for women [1]. Germline pathogenic variants (PVs) in *BRCA1* or *BRCA2* (*gBRCA1/2*) are observed in 3% of unselected BC and in 10–15% of patients with triple-negative BC (TNBC) [2]. BRCA1 and BRCA2 play an essential role in homologous recombination (HR), the DNA damage repair pathway that allows DNA double-strand breaks (DSBs) to be repaired in an error-free manner [3]. Next to *BRCA1* and *BRCA2,* PVs in other HR-related genes such as *PALB2*, *RAD51C* and *RAD51D* as well as epigenetic silencing of *BRCA1* and *RAD51C* via hypermethylation of the gene promotor have been shown to lead to HR deficiency (HRD) [4]. Overall, HRD can be observed in approximately 18% of BC [4], a substantial group of patients who could potentially benefit from treatment with platinum-based chemotherapy or poly-ADP ribose polymerase (PARP) inhibitors [5, 6]. At this moment, however, PARP inhibitors (PARPi) are only approved for the treatment of patients with *gBRCA1/2* or suspected *gBRCA1/2*, human epidermal growth factor receptor type 2 (HER2)-negative BC with early (olaparib) or recurrent disease (olaparib and talazoparib) [7–10]. Various efforts are currently undertaken to develop DNA-based and functional HRD tests to identify an additional group of BC patients with HRD tumors who might benefit from treatment with PARPi [11].

DNA-based HRD tests comprise those identifying PVs in HR-related genes, mutational signatures, and/or genomic scars by next-generation sequence (NGS) analysis of tumor DNA [12–15]. Currently, several clinical studies are ongoing to determine the accuracy of DNA-based HRD tests to predict platinum and/or PARPi response [12, 16, 17]. The myChoice HRD test, in which a score is calculated based on genomic loss of heterozygosity (LOH), telomeric allelic imbalance (TAI), and large-scale transition (LST), was shown to be a good predictor for PARPi sensitivity in ovarian cancer (OC) patients [18, 19]. The myChoice HRD test did not, however, predict carboplatin sensitivity in TNBC patients [20]. Alternatively, HRDetect, which applies both mutational signature and genomic scar analysis, was predictive for rucaparib response in a prospective clinical trial with TNBC patients [21]. Importantly, patient selection based on DNA-based HRD tests is still suboptimal, as PARPi and platinum benefit are also observed among patients with non-HRD tumors [20, 22].

In RAD51-based functional HRD tests, the ability of tumor cells to accumulate RAD51 protein at sites of DNA damage in proliferating (geminin-positive, GMN^+^) tumor cells is assessed [23–27]. RAD51 scores (i.e., the percentage of RAD51^+^/GMN^+^ cells) are used as a functional HR read-out in tumor samples, where low RAD51 scores indicate HRD. The first developed RAD51-based HRD test, the REcombination CAPacity (RECAP) test, has shown a high sensitivity in identifying breast and ovarian tumors with PVs in *BRCA1/2* or *BRCA1* promoter hypermethylation [23, 26, 28–31]. However, the requirement for fresh tumor tissue poses a significant limitation for its clinical implementation. To address this limitation, the RAD51-FFPE test has been introduced as a more practical alternative. Unlike the RECAP test, the RAD51-FFPE test utilizes FFPE diagnostic tumor samples, eliminating the need for fresh tumor tissue [27]. The RAD51-FFPE test parameters were established based on its sensitivity to identify i) tumors with *BRCA1/2* PVs, and ii) tumors identified as HRD using the functional RECAP HRD test on ovarian and endometrial tumors [27].

Although sample sizes were small, two studies have demonstrated a correlation between low RAD51-FFPE scores and sensitivity to platinum or PARPi in metastatic BC and TNBC patients, respectively [32, 33]. In a recent biomarker analysis from the GeparSixto trial, low RAD51-FFPE scores exhibited a strong association with the presence of *BRCA1/2* PVs and myChoice HRD, accurately predicting the clinical benefit of adding carboplatin to neoadjuvant chemotherapy (NACT) treatment in TNBC [34]. Another study, evaluating PARPi sensitivity in over 100 patient-derived xenograft models from BC, showed that low RAD51-FFPE scores displayed a higher accuracy in predicting PARPi response compared to HR gene mutations and genomic HRD analysis, including both myChoice HRD and HRD signature assessment by HRDetect [35].

The accuracy of the RAD51-FFPE test to identify ‘true’ HRD samples in BC remains uncertain. In this study, we evaluate the performance of the RAD51-FFPE test using previously defined test parameters for ovarian and endometrial cancer. Our findings demonstrate that the RAD51-FFPE test can achieve a high sensitivity in identifying *BRCA-*deficient and RECAP-HRD samples in BC, regardless of the histological subtype.

## Materials and methods

### Patient material

Archival diagnostic FFPE tumor tissue blocks of BC patients who underwent surgery at the Leiden University Medical Center (LUMC) were collected between May 2013 and August 2019. The selection was made based on the availability of matched, cryopreserved tumor tissue that could be used for the RECAP test. All samples were coded with a unique research code. The local medical ethics committee of the LUMC approved the study protocols on 7 February 2011 and 24 May 2017 (P10.226, B16.019, G17.041) and samples were handled according to the “Code for Proper Secondary Use of Human Tissue “ in the Netherlands as established by the Dutch Federation of Medical Scientific Societies.

### γH2AX/GMN co-immunohistochemistry staining (co-IHC)

Tissue sections were stained for γH2AX (mouse, monoclonal, MilliporeSigma, St. Louis, MO, U.S., cat. 05-636, clone JBW301) and GMN (rabbit, polyclonal, Proteintech, Manchester, U.K., cat. 10802-1-AP) according to a previously published protocol [27].

### RAD51/GMN co-immunofluorescence staining (co-IF)

Tissue sections were stained for DAPI, RAD51 (rabbit, monoclonal, Abcam, Cambridge, U.K., cat. ab133534), and GMN (mouse, monoclonal, NovoCastra, Leica Biosystems, Buffalo Grove, IL, U.S., cat. NCL‐L) according to a previously published protocol [27].

### Sample selection

Based on the availability of cryopreserved BC tumor samples, representative matching diagnostic FFPE blocks containing > 70% vital tumor tissue were selected by a mamma pathologist (D.C.). Samples with ductal carcinoma in situ (DCIS) were not included in this study and pleural-fluid samples were additionally screened for the presence of p53 mutant cells based on IHC staining to confirm the presence of tumor cells.

Next, the presence of sufficient GMN^+^ cells was confirmed based on a GMN/RAD51 co-IF for both RECAP and RAD51-FFPE test samples. At least 40 GMN^+^ cells, randomly selected in 3–5 vital tumor tissue areas, were considered sufficient. Tumor samples with < 40 GMN^+^ cells in the co-IF were excluded for analysis.

For RAD51-FFPE test samples, the presence of endogenous DNA damage in tumor cells of FFPE samples with a RAD51-FFPE score of ≤ 15% (Sect. “RAD51-FFPE test”) was confirmed by evaluation of a γH2AX/GMN co-IHC. At least 40 GMN + cells, randomly selected in 3–5 vital tumor tissue areas, were manually counted by two independent observers on a Zeiss Axio Imager.M2 light microscope, 63 × oil objective. The number of γH2AX foci were counted per selected GMN^+^ cell (0, 1, 2, 3, 4, or ≥ 5 γH2AX foci). The γH2AX score was determined by calculation of the average percentage of γH2AX^+^/ GMN^+^ cells (cut-off ≥ 2 γH2AX foci) of two observers. Diagnostic FFPE tumor samples with a γH2AX score < 25% were excluded for analysis due to the absence of sufficient endogenous DNA damage.

### RAD51-FFPE test

Diagnostic FFPE tumor tissue sections were stained for DAPI, GMN, and RAD51 in a co-IF staining and scored manually with a Leica DM6B microscope, 63×/1.40–0.6 oil objective with an EL6000 light source. DAPI was used to get an overall impression of the sample (either whole tumor section or pleural fluids enriched for tumor cells), assess cell morphology and locate 3–5 areas of the sample enriched with vital tumor cells. Within vital tumor areas, GMN^+^ cells were identified and ≥ 40 GMN^+^ cells were selected at random. A cell was considered GMN^+^ when the nucleus was completely stained with a granular pattern. The number of RAD51 foci within a GMN^+^ cell was determined (0, 1, 2, 3, 4, or ≥ 5 foci) and cells were categorized accordingly. For each RAD51 foci cut-off, a RAD51-FFPE score was calculated as the percentage of RAD51^+^/GMN^+^ cells by each observer. Final RAD51-FFPE scores were calculated as the average RAD51-FFPE score of two independent observers.

### RECAP test

A detailed description of the methodology of the RECAP test has previously been published [26]. In short, tumor samples (thawed after cryopreservation), were irradiated with ionizing radiation to induce DNA DSBs and incubated at 37°C for two hours prior to fixation and paraffin embedding. Irradiated tumor samples with high tumor percentage and sufficient tumor vitality (see quality description in Sect. "Sample selection") were included and stained for GMN (anti-geminin antibody, ProteinTech, Manchester, U.K., cat. 10802–1-AP) and RAD51 (anti-RAD51 antibody, GeneTex, Alton Pkwy Irvine, CA, U.S., cat. GTX70230) with a co-IF staining. Forty GMN^+^ cells were evaluated for the presence of ≥ 5 foci/nucleus (RAD51^+^). The percentage of RAD51^+^/GMN^+^ cells was represented as the RECAP score. Tumor samples were considered HR-Deficient (HRD) with a RECAP score of ≤ 20%, HR-Intermediate (HRI) with a RECAP score of 21–50% and HR-Proficient (HRP) with a RECAP score of > 50%.

### Genetic and epigenetic analyses

NGS analysis was performed using maximum 30 ng of tumor DNA per sample isolated from FFPE tissue blocks. The mean tumor cell percentage of included samples was 61% (range: 10–80%). All samples were sequenced with an HRD targeted gene panel. The custom Ampliseq HRDv2 gene panel (SeqStudio Genetic Analyzer, Thermo Fisher Scientific) was used for variant detection in the coding exons of the following genes: *ATM* (exon 2-63), *BARD1* (exon 1-11), *BRCA1* (exon 2-24), *BRCA2* (exon 2-27), *BRIP1* (exon 2-20), *CDK12* (exon 1-14), *CHEK1* (exon 2-13), *CHEK2* (exon 2-15), *FANCL* (exon 1-14), *PALB2* (exon 1-13), *PPP2R2A* (exon 1-10), *RAD51B* (exon 2-11), *RAD51C* (exon 1-9), *RAD51D* (exon 1-10), *RAD54L* (exon 1-18), *TP53* (exon 1-11), *PIK3CA* (hotspots in exon 2, 5, 7, 8, 10, 14, 19, and 21), and *ERBB2* (hotspots in exon 8 and 17-21). Details on request (DT). Sequencing was performed in an Ion GeneStudio S5 Series sequencer (ThermoFisher Scientific). The raw, unaligned sequencing reads were mapped against human reference genome (hg19) using TMAP software. Torrent Variant Caller was used for variant calling and variants were categorized using the 5-tier pathogenicity classification according to Plon *et al*.: Class 1 = benign, Class 2 = likely benign, Class 3 = variant of uncertain significance (VUS), Class 4 = likely pathogenic, and Class 5 = pathogenic [36]. When needed, variants were interpreted using Integrative Genomic Viewer or the Alamut™ Visual Plus software (SOPiA GENETICS ™. Only class 3, 4, and 5 variants are reported in this manuscript. LOH analysis of the NGS data was performed as described previously by de Jonge *et al*. [37].

In addition to sequence analysis, promoter hypermethylation of *BRCA1* using MS-MLPA was measured on samples for which sufficient DNA was available, as described previously [37].

### Statistical analysis

Figures were created with Graphpad Prism 8.0 (GraphPad Software, San Diego, CA, U.S.), Adobe Illustrator CC 2020 (Adobe Inc, San Jose, CA, U.S.), and BioRender software (Toronto, ON, Canada). Statistical analysis was performed with Graphpad Prism 8.0, IBM SPSS version 25.0 (SPSS Inc.), and SigmaStat 3.5 (Systat Software Inc, San Jose, CA, U.S.). Student’s *t* tests were performed to test differences between two groups containing normally distributed numerical data and Mann–Whitney Rank Sum tests when numerical data was not normally distributed. Categorical data of two groups were tested with Chi-square test or Fisher’s Exact test. Fisher’s Exact test was chosen when at least one of the expected values was less than one and when over 20% of the expected values were less than five. To test if numerical data was correlated between two groups, Pearson’s correlation coefficient was calculated. A *p* value of < 0.05 was considered significant.

## Results

### RAD51-FFPE test

**Fig. 1:**
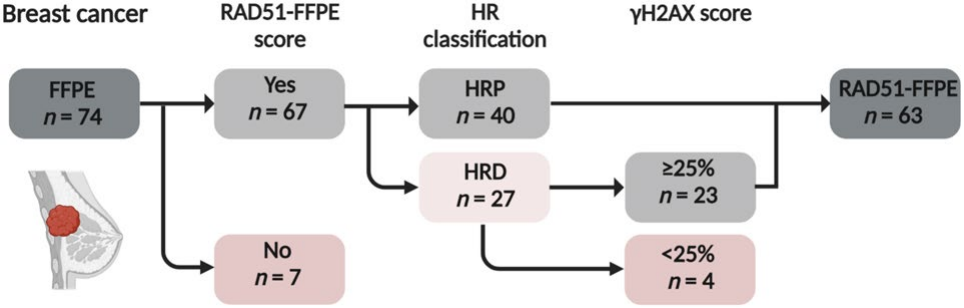
Flowchart for the inclusion of RAD51-FFPE BC samples. In total, 74 breast cancer samples were analyzed with the RAD51-FFPE test. Sixty-seven samples contained sufficient geminin-positive (GMN^+^) tumor cells to allow assessment of a RAD51-FFPE score. Twenty-seven samples were initially classified as HRD, but four samples were excluded from the cohort as they contained insufficient levels of endogenous DNA damage. In total, 63 samples were included in the RAD51-FFPE study cohort. There were no significant differences in patient and tumor characteristics between included and excluded samples (Suppl. Table 1). *FFPE* formalin-fixed paraffin-embedded, *HRP* homologous recombination proficient, *HRD* homologous recombination deficient

A cohort of 74 diagnostic FFPE BC samples was selected based on the availability of cryopreserved tumor tissue. The RAD51-FFPE score in these samples was determined by analyzing the accumulation of RAD51 protein at sites of DNA damage in proliferating tumor cells [27]. In seven samples, the number of GMN^+^ cells was insufficient to determine a RAD51-FFPE score (Fig.1).

Out of the 67 tumor samples for which a final RAD51-FFPE score was determined, 27 samples were categorized as HRD. For these samples, an additional GMN/γH2AX staining was performed to confirm that the low RAD51-FFPE score was not caused by insufficient levels of endogenous DNA damage (Suppl. Figure 1). Four samples (Two no special type grade 3, one lobular grade 2, and one papillary carcinoma grade 1) with an γH2AX score (percentage of GMN^+^ cells showing at least two γH2AX foci) of < 25% were excluded (Fig. 1, Suppl. Figure 2, Materials and Methods Sect. "Sample selection"). In total, the HR status was successfully determined for 63 out of the 74 (85%) BC samples (Suppl. Table 2), with 37% (23/63) being classified as HRD (Fig. 1).

### (Epi) genetic defects in HR-related genes in relation to RAD51-FFPE scores

To assess the sensitivity of the RAD51-FFPE test to identify breast tumors with genomic-HRD, i.e., (epi)genetic defects in HR-related genes, we performed NGS analysis applying an HRD gene panel comprising 18 genes and applied MS-MLPA to identify *BRCA1* promotor hypermethylation (Materials and Methods Sect. "Genetic and epigenetic analyses").

**Fig. 2:**
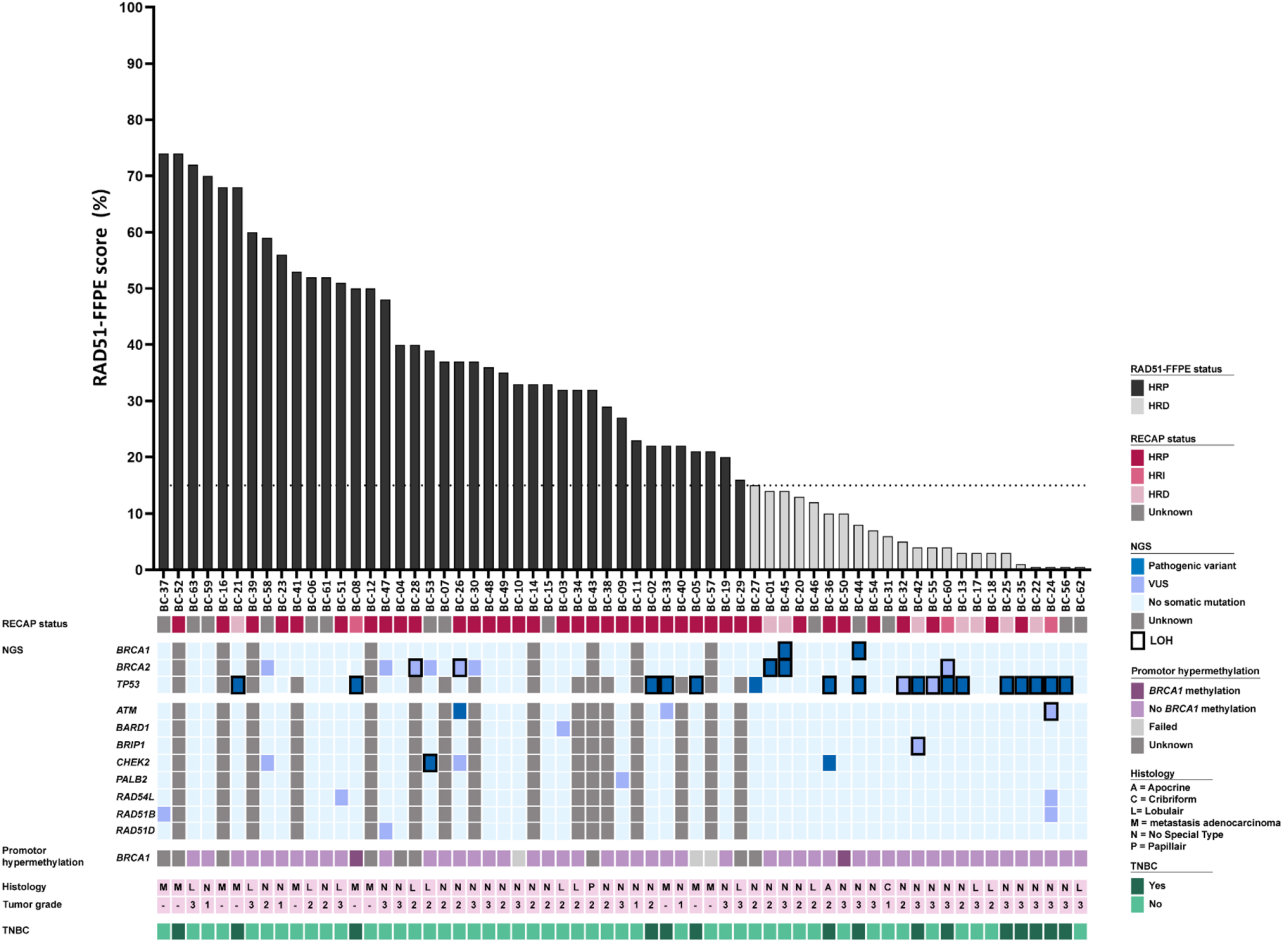
RAD51-FFPE score in relation to RECAP status, HR gene panel results, *BRCA1* promotor hypermethylation status, and tumor characteristics. RAD51-FFPE scores were calculated as the percentage of geminin-positive (GMN^+^) cells with ≥ 2 RAD51 foci. *HRP* homologous recombination proficient, *HRI* homologous recombination intermediate, *HRD* homologous recombination deficient, *RECAP* REcombination CAPacity, *LOH* loss-of-heterozygosity, *VUS* variant of uncertain significance, *NGS* next-generation sequencing, *TNBC* triple-negative breast cancer

A PV in *BRCA1* and/or *BRCA2* with LOH of the wild-type allele was identified in three out of 23 HRD tumors, providing an explanation for the observed HRD phenotype (i.e., BC-01 (*BRCA2* PV), BC-44 (*BRCA1* PV), and BC-45 (*BRCA1* and *BRCA2* PV), Fig. 2, Suppl. Table 3)). In one HRD sample, a *CHEK2* PV with a variant allele frequency (VAF) of 0.46 was identified (BC-36). One HRD tumor harbored a VUS with a VAF ≥ 0.5 in *BRIP1* with LOH of the wild-type allele (BC-42; Fig. 2, Suppl. Table 3). Two other HRD tumors harbored a VUS in *ATM* and *BRCA2* with LOH of the wild-type alleles, respectively, but both had a VAF < 0.5 (BC-24 and BC-60; Fig. 2, Suppl. Table 3).

Among the 40 samples that were classified as HRP, no PVs were identified in *BRCA1* or *BRCA2*. In one sample, a *CHEK2* PV with a VAF ≥ 0.50 and LOH of the wild-type allele was identified (BC-53). A PV in *ATM* with a VAF < 0.5 was identified (BC-26). Various VUSes in *ATM*, *BRCA2*, *CHEK2*, *RAD51B*, and *RAD54L* were identified among the HRP samples, of which five had a VAF ≥ 0.50 (Fig. 2, Suppl. Table 3).

All genetic variants identified are listed in Suppl. Table 3. *BRCA1* promotor hypermethylation was identified in one HRP (BC-08) and in one HRD sample (BC-50).

In conclusion, the sensitivity of the identification of BC samples with a PV in *BRCA1* and/or *BRCA2* was 100% in our cohort.

### The RAD51-FFPE test identifies RECAP-HRD BC samples with high sensitivity

To determine the sensitivity and specificity of the RAD51-FFPE test to identify functional HRD, we performed the RECAP test on viable, cryopreserved, matching tumor samples on the premise that the HRD status as determined by the RECAP test faithfully represented the functional HR status.

#### The RECAP test

**Fig. 3:**
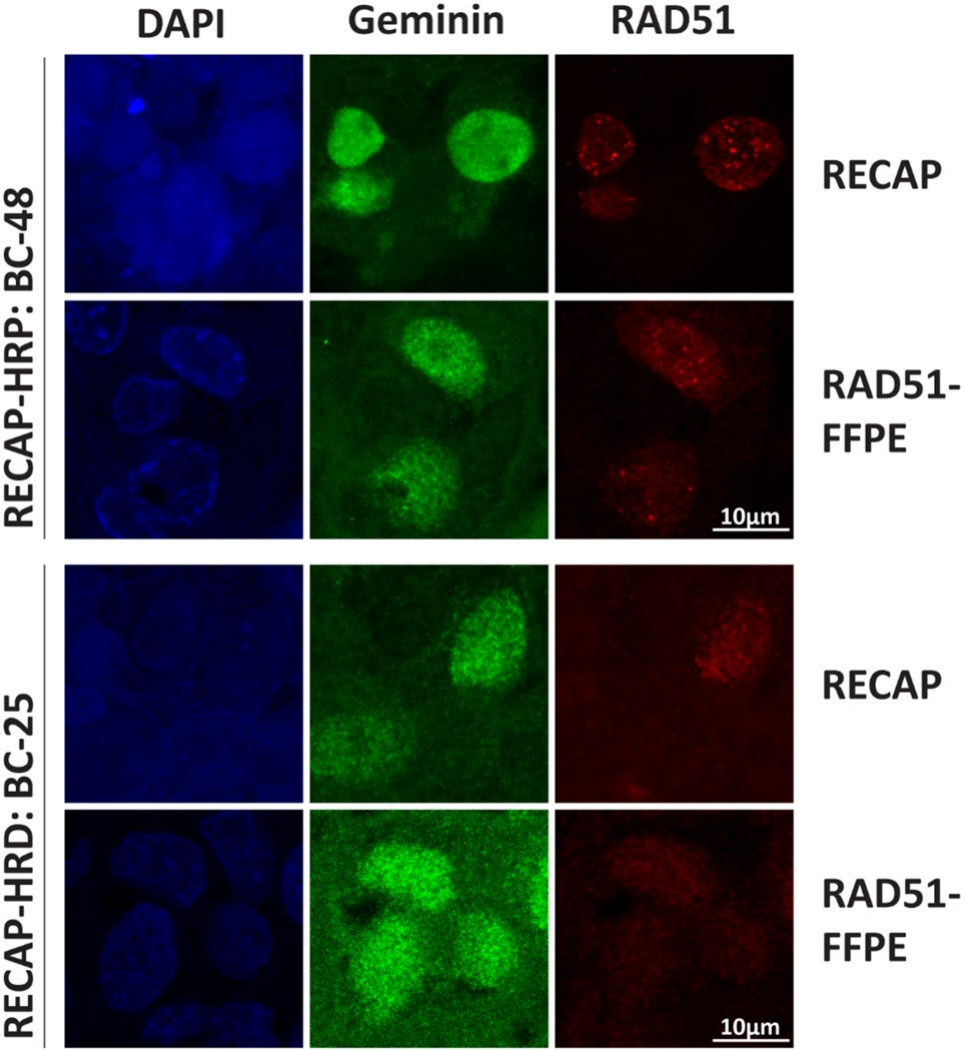
Microscopy illustration of RECAP and diagnostic FFPE co-IF slides of an homologous recombination proficient (HRP) and homologous recombination deficient (HRD) BC. *BC* breast cancer, *RECAP* REcombination CAPacity, *FFPE* formalin-fixed paraffin-embedded, *HRP* homologous recombination proficient, *HRD* homologous recombination deficient

The RECAP test is a RAD51-based functional HRD test using viable tumor tissue for the identification of HRD in BC [23, 28, 30, 31]. In contrast to the RAD51-FFPE test, tumor tissue is irradiated with 5 Gy ionizing radiation to induce DNA damage prior to fixation. As we had access to cryopreserved tumor tissue from the 63 BC samples in our cohort with informative RAD51-FFPE scores, we determined RECAP scores on matched tumor samples. Quality control steps involved assessment of tissue quality to determine tissue vitality and the presence of ≥ 40 GMN^+^ tumor cells [26] (Sect. “RECAP test”). Out of 63 BC samples, a total of 14 samples were excluded based on these criteria (Suppl. Figure 3). In total, RECAP scores were determined for 49/63 (78%) BC samples, of which eight (16%) were classified as HRD, three (6%) as HR-Intermediate (HRI), and 38 (78%) as HRP (Fig. 2, Suppl. Figure 3). Figure 3 shows representative co-IF images of HRP and HRD samples from matched RECAP and RAD51-FFPE samples.

#### The correlation between RECAP and RAD51-FFPE scores

**Fig. 4:**
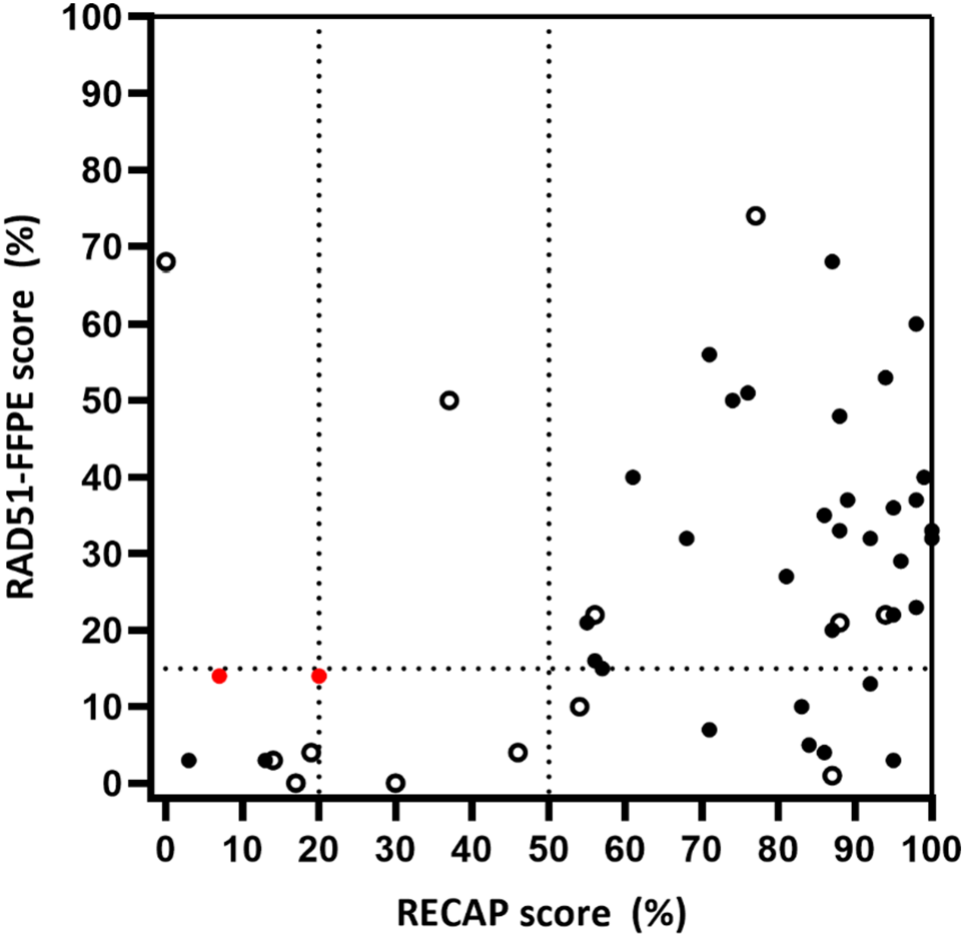
RECAP versus RAD51-FFPE scores (*n* = 49). RAD51-FFPE scores were determined as the percentage of geminin-positive cells with at least two RAD51 foci. RECAP scores were determined as the percentage of geminin-positive cells with at least five RAD51 foci. HRD thresholds for the RAD51-FFPE test and RECAP test are indicated with dashed lines. Samples with a pathogenic variant in *BRCA1* and/or *BRCA2* are indicated in red. TNBC samples are indicated with open circles. *FFPE* formalin-fixed paraffin-embedded, *RECAP* REcombination CAPacity, *TNBC* triple-negative breast cancer

For 49 BC samples both RAD51-FFPE and RECAP scores were obtained (Figs. 2 and 4). The RAD51-FFPE test showed a high sensitivity to identify HRD BC samples as defined by the RECAP test (RECAP-HRD) as seven out of the eight RECAP-HRD BC samples were identified as HRD by the RAD51-FFPE test, including two samples with a *BRCA1/2* PV (Figs. 2 and 4, Suppl. Table 4). One RECAP-HRD sample (BC-21, RECAP score 0%) was scored as HRP (68%) by the RAD51-FFPE test (Fig. 4, Suppl. Table 2).

For the 38 BC samples classified as RECAP-HRP, 29 samples were classified as HRP by both tests. Nine samples were classified as HRD by the RAD51-FFPE test (Figs. 2 and 4, Suppl. Table 4).

Using the test parameters previously established for OC and EC (HRD threshold of 15% with a RAD51-FFPE foci cut-off of two), the RAD51-FFPE test demonstrated an 88% sensitivity and 76% specificity in identifying the same HR-class as defined by the RECAP test [27]. Adjusting the RAD51 foci cut-off and/or the percentage of RAD51^+^/GMN^+^ cells to define HRD did not improve the sensitivity and specificity of the RAD51-FFPE test (Suppl. Figure 4 and Suppl. Table 5).

The three RECAP-HRI samples were not taken along for the calculation of the sensitivity and specificity of the RAD51-FFPE test as the RAD51-FFPE test does not have an HRI scoring class.

### HRD is commonly identified among TNBC and *TP53* mutated breast cancers

Our cohort consisted of a heterogenous set of BC samples with different histologic subtypes (Suppl. Table 2). To investigate if HRD is more prevalent in specific tumor types, we stratified clinicopathologic characteristics by HR-status as determined by the RAD51-FFPE test (Table 1). Although not statistically significant, HRD tumors were more often observed in TNBC, 9/15 (60%) and in *TP53* mutated BC, 12/17 (71%) (Table 1).

**Table 1:** Clinicopathologic characteristics stratified for HR status as determined by the RAD51-FFPE test

	HRD* (*n* = 23) *n* (%)	HRP* (*n* = 40) *n* (%)	*P* value
Age at diagnosis (Years ± SEM)	67.9 (± 3.09)	61.7 (± 1.98)	0.080
Tumor			**0.010**^**a**^
Primary	23 (100)	30 (75)	
Recurrent		10 (25)	
Histologic subtype			0.531^b^
No special type (NST)	17 (74)	20 (67)	
Lobular	4 (17)	9 (30)	
Other			
* Papillary*		1 (3)	
* Apocrine*	1 (4)		
* Cribriform*	1 (4)		
Tumor grade			0.141^c^
1	1 (4)	4 (10)	
2	8 (35)	15 (38)	
3	14 (61)	11 (28)	
NA		10 (25)	
Hormone receptor status			0.063^d^
TNBC	9 (39)	6 (15)	
Other			
* ER* + */PR* + */Her2Neu-*	11 (48)	23 (58)	
* ER* + */PR* + */Her2Neu* +	1 (4)	3 (8)	
* ER* + */PR-/Her2Neu-*	2 (8)	4 (10)	
* ER* + */PR-/Her2Neu* +	–	3 (8)	
* ER-/PR-/Her2Neu* +	–	1 (3)	
*TP53* PV			0.053^e^
Yes	12 (52)	5 (21)	
No	11 (48)	19 (79)	
*BRCA1/2* PV			0.068^e^
Yes	3 (13)		
No	20 (87)	32 (100)	
*BRCA1* promotor hypermethylation			1.000^a^
Yes	1 (5)	1 (3)	
No	21 (95)	28 (97)	

Differences between the HRD and HRP groups were statistically tested with a *t* test for the age at diagnosis and the chi-square or Fisher’s Exact test for the other characteristics. Significant *p* values are indicated in bold

*NA* not applicable, *HRD* homologous recombination deficient, *HRP* homologous recombination proficient, *PV* pathogenic variant

^*^Due to rounding corrections, the total percentage is not always 100%. ^a^ Fisher’s exact. ^b^Chi-square, ‘NST vs ‘lobular’. ^c^Chi-square, ‘grade 1–2’ vs ‘grade 3’. ^d^Chi-square, ‘TNBC’ vs ‘other’. ^e^Chi-square

No differences were observed in tumor grade and age at diagnosis between the HRD and HRP groups (*p* = 0.141 and *p* = 0.080, respectively, Table 1). The tumor samples derived from pleural fluid at recurrent disease (*n* = 10) were all classified as HRP (*p* = 0.010, Table 1). No differences in clinicopathologic characteristics between included and excluded RAD51-FFPE samples were observed (Suppl. Table 1).

Stratification of clinicopathologic characteristics based on HR group classification by the RECAP test displayed only a significant difference between HRD and HRP groups for the presence of *BRCA1/2* PVs (Suppl. Table 6).

## Discussion

Here, we determined the sensitivity and specificity of the RAD51-FFPE test in a diagnostic series of BC using the ex vivo RAD51-based HRD test (RECAP test) and *BRCA*-deficiency as gold standards for HRD classification.

In this study, RAD51-FFPE scores were successfully determined for 63 BC samples. Thirty-seven percent of the BC samples in our cohort was identified as HRD using the RAD51-FFPE test, including three tumors with a *BRCA1/2* PV. The prevalence of HRD was higher among TNBC (60%) and tumors with *TP53* PVs (71%), which is in line with results obtained in other studies evaluating the prevalence of HRD with a RAD51-based HRD test on diagnostic TNBC tumor samples [21, 33, 38].

Two samples harbored a pathogenic variant in *CHEK2*, one with LOH of the wild-type allele, classified as HRP (BC-53) and one with unknown LOH status of the wild-type allele that was classified as HRD (BC-36). These results are in line with findings indicating that loss of CHK2 activity does not lead to HRD [39, 40]. Similarly, breast tumors with *ATM* PVs lack HRD-related mutational signatures, in line with our functional classification of sample BC-26 with an *ATM* PV as HRP [41]. In six BC samples, VUSes with a VAF ≥ 0.50 were identified in *ATM*, *BRIP1*, *CHEK2*, *BRCA2*, *RAD51B*, and *RAD54L* with LOH of the wild-type allele in two samples. The c.2240A > G, p.(Glu747Gly) VUS in *BRCA2* is located outside the known functional domains and has been reported not to affect protein function, in line with the observed HRP phenotype of the tumor [42]. One HRD sample harbored a *BRIP1* VUS c.2768 T > G, p.(Leu923Arg) with LOH of the wildtype allele. Based on the available information, it is unlikely that this variant can explain the HRD phenotype observed in the sample. Not only is the effect of the missense variant on BRIP1 activity yet unknown, previous research in cell lines showed that *BRIP1*-deficiency does not impair RAD51 foci formation [43].

The RAD51-FFPE test correctly identified the three *BRCA1/2*-deficient tumors present in our set as HRD. The sensitivity for the identification of functional HRD as defined by the RECAP test was 88% with a specificity of 76% using previously validated thresholds for OC and EC [27]. The observed frequency of 16% HRD in our RECAP analyses is in line with previously reported RECAP results in a cohort of 125 BC [23]. Using the RAD51-FFPE test, we observed a higher frequency of HRD samples compared to the RECAP test (Suppl. Table 4). It is important to realize that there are differences in the nature and quantity of DNA DSBs and the size of the RAD51 foci between the endogenous (RAD51-FFPE) and radiation-induced (RECAP) DNA damage. In addition, it cannot be ruled out that time-to-fixation might play a role as has previously been described for HER2 assessment [44, 45]. While the fixation period is exactly two hours for all RECAP samples, it varies considerably for the RAD51-FFPE samples due to their diagnostic nature. If the detection of RAD51 foci is compromised in samples with suboptimal time-to-fixation (potentially affecting the immune reactivity of the protein), this might explain the false positive HRD samples observed. However, further investigation is required to explore this possibility.

Given the high incidence of BC and the relatively low frequency of PVs in *BRCA1* or *BRCA2* in these carcinomas, there is a clear demand for a sensitive method to enrich for HRD tumors in a fast and cost-effective manner. The availability of a sensitive, non-DNA-based, method like the RAD51-FFPE test addresses this need and fills an important gap in the diagnostic landscape as, in contrast to the RECAP test, no ex vivo irradiated fresh tumor tissue is required. Although the RAD51-FFPE test may overestimate the number of ‘true’ HRD samples, it faithfully captured all *BRCA-*deficient samples. It therefore offers a reliable and efficient method with a success rate of more than 90% to identify HRD tumors even in situations where DNA-based testing is challenging or not readily accessible in routine clinical practice. This is particularly important considering the potential implications for treatment decisions and patient stratification.

### Supplementary Information

Below is the link to the electronic supplementary material.

Supplementary file1 (PDF 750 KB)

## Data Availability

All data generated or analyzed during this study are included in this published article (and its supplementary information files).
