# Supplementary material for: Performance of a RAD51-based functional HRD test on paraffin-embedded breast cancer tissue
Source: Breast Cancer Res Treat. 2023 Sep 19;202(3):607–16. doi: 10.1007/s10549-023-07102-y (PMC10564840; doi:10.1007/s10549-023-07102-y)
Supplement: Supplementary file 1 — Supplementary file1 (PDF 750 KB) [file 10549_2023_7102_MOESM1_ESM.pdf]

Supplementary Material

Breast Cancer Research and Treatment

Performance of a RAD51-based functional HRD test on paraffin-embedded breast cancer tissue

Lise M. van Wijk [1], Sylvia Vermeulen[1], Natalja T. ter Haar[2], Claire J.H. Kramer[2], Diantha Terlouw[2], Harry Vrieling[1], Danielle Cohen[2], and Maaike P.G. Vreeswijk[1*]

1 Department of Human Genetics, Leiden University Medical Center, 2300 RC Leiden, The Netherlands.
2 Department of Pathology, Leiden University Medical Center, 2300 RC Leiden, The Netherlands.

*Correspondence to Maaike Vreeswijk, M.P.G.Vreeswijk@lumc.nl

Content

Additional analyses

Adjustments of RAD51 foci cut-off and HRD threshold to define HRD in BC.

Supplementary Figures

Suppl. Fig. 1 - Immunohistochemical slides of tumors with a low and high γH2AX score.

Suppl. Fig. 2 - γH2AX scores of RAD51-FFPE HRD samples.

Suppl. Fig. 3 - Flowchart for the inclusion of RECAP samples.

Suppl. Fig. 4 - ROC curves for RAD51-FFPE scores with a RAD51 foci cut-off ranging from 1 till 5.

Supplementary Tables

Suppl. Table 1 - Comparison of clinicopathologic characteristics between included and excluded RAD51-FFPE BC samples.

Suppl. Table 2 - Overview tumor characteristics informative study cohort (*n* = 63).

Suppl. Table 3 - Overview genetic variants identified in our BC cohort.

Suppl. Table 4 - HR classification by the RECAP and RAD51-FFPE tests.

Suppl. Table 5 - Sensitivity and specificity analysis for the RAD51-FFPE test with the RECAP test serving as gold standard.

Suppl. Table 6 - Clinicopathologic characteristics stratified for HR status as determined with the RECAP test.

Supplementary Information

Additional analyses

Adjustments of RAD51 foci cut-off and HRD threshold to define HRD in BC.

The HRD threshold and RAD51 foci cut-off for the evaluation of HR status of breast FFPE samples has been previously described at 10% with a RAD51 foci cut-off of five [24, 25, 33-35], while an HRD threshold of 10% and 20% with a RAD51 foci cut-off of one and five respectively, was applied when biopsies were analyzed from TNBC patients after receiving neoadjuvant chemotherapy or rucaparib treatment [21, 47]. Since our BC cohort was heterogeneous, including tumor samples with different histologic subtypes and treatment history (Suppl. Table 2), we explored whether changing the RAD51-FFPE test parameters would lead to an increase in sensitivity and/or specificity. A sensitivity and specificity analysis for commonly described HRD thresholds (5,10, 15, 20%) and RAD51 foci cut-offs (1, 2, 3, 4 or 5) showed that the highest sensitivity and specificity of 88% and 76% respectively was reached with an HRD threshold of 5% with a RAD51 foci cut-off of four, and with an HRD threshold of 15% and a RAD51 foci cut-off of two, as applied in our study (Suppl. Table 5).

To investigate whether alternative, not previously described HRD thresholds, would reach a better sensitivity and specificity using RAD51-FFPE scores to classify tumors with the RECAP outcome as gold standard, Receiver Operating Characteristic (ROC) curves were plotted and HRD thresholds were applied ranging from 0-100% with a 1% step size (Suppl. Fig. 4). Importantly, RAD51-FFPE foci cut-offs of 2, 3 and 4 led to a very good performance with area under the curve (AUC) scores > 0.8 (0.804, 0.814, and 0.817 respectively). The highest sensitivity and specificity was reached when applying an HRD threshold of 7% with a RAD51 foci cut-off of three, leading to a sensitivity of 88% with a specificity of 84% (Suppl. Fig. 4C). The second best test parameters were an HRD threshold of 15% with a RAD51 foci cut-off of two, a 5% HRD threshold with a RAD51 foci cut-off of four, and a 4% HRD threshold with a RAD51 foci cut-off of five, all leading to a sensitivity of 88% with a specificity of 79% for the identification RECAP-HRD. All RAD51-FFPE test parameter combinations led to a 100% sensitivity for the identification of samples with a *BRCA1/2* PV.

Supplementary Figures

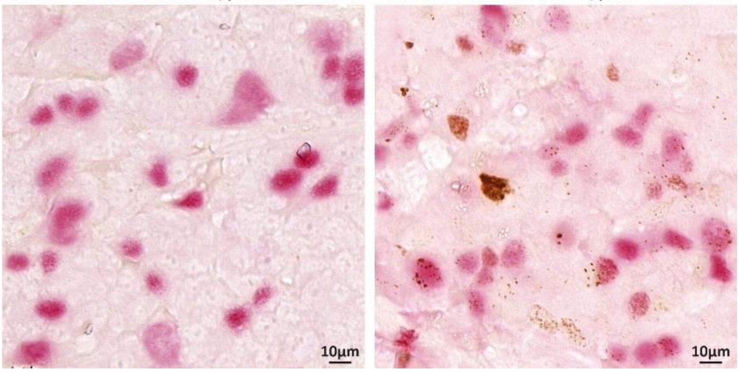

Suppl. Fig. 1 Immunohistochemical slides of tumors with a low and high γH2AX score. Geminin (GMN, pink)/γH2AX, brown) immunohistochemical slides of a tumor with a γH2AX score <25% (left) and ≥25% (right).

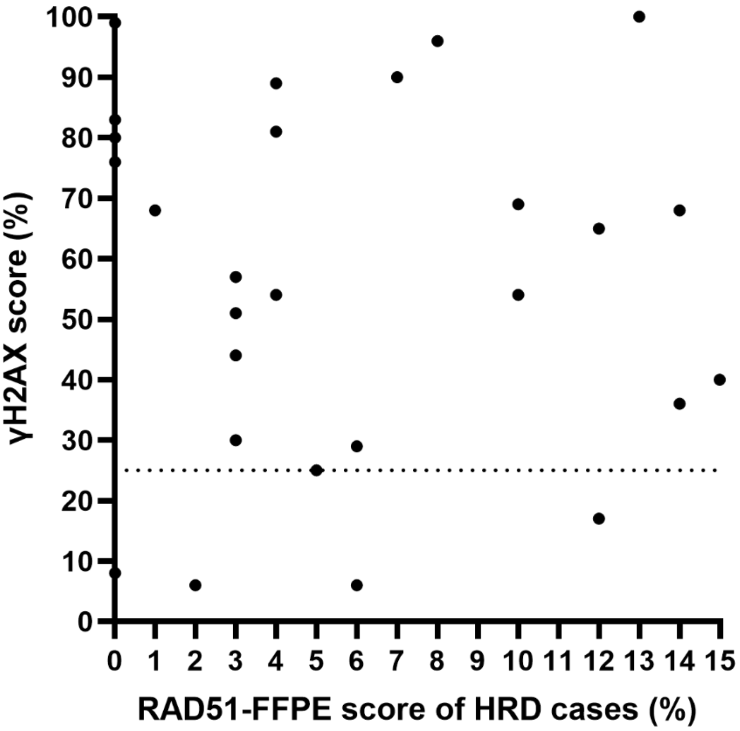

Suppl. Fig. 2 γH2AX scores of RAD51-FFPE HRD samples. In total, 27 samples had a RAD51-FFPE score ≤15% and were stained for GMN/γH2AX by IHC and γH2AX scores (% GMN⁺ cells with ≥2 γH2AX foci) were calculated**.** Samples with a γH2AX score <25%, were excluded from analysis. Twenty-three samples had a γH2AX score ≥25%. Abbreviations: FFPE = formalin-fixed paraffin-embedded, HRD = homologous recombination deficient, GMN = geminin.

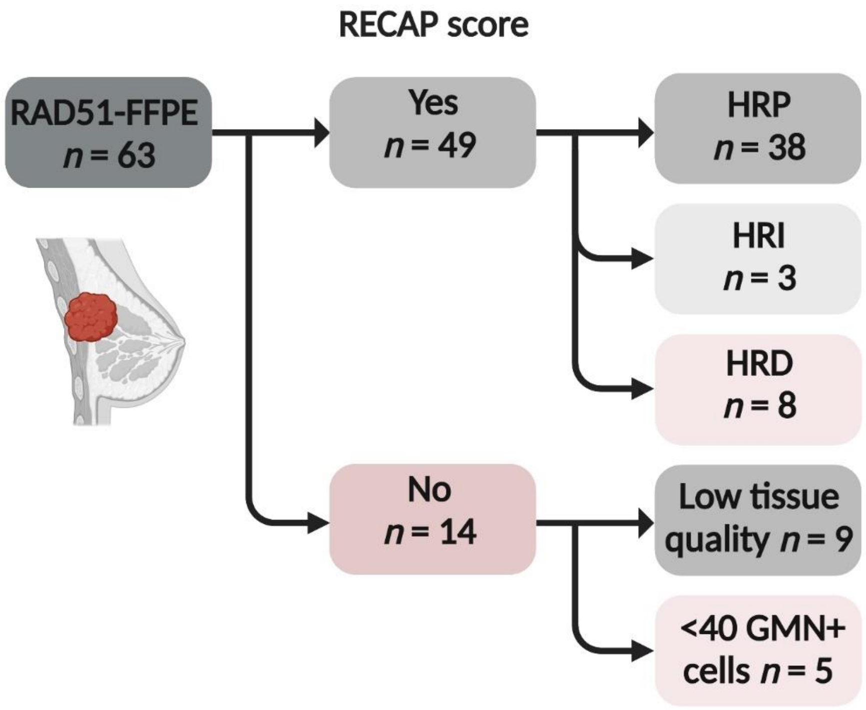

Suppl. Fig. 3 Flowchart for the inclusion of RECAP samples. For the 63 FFPE samples with an informative RAD51-FFPE score, the RECAP test was performed using the available cryopreserved tumor tissue. RECAP scores were successfully calculated for 49 samples. For 14 samples, no RECAP scores were calculated due to insufficient tissue quality (no vital tissue and/or a low tumor cell percentage) or due to a low number of geminin-positive (GMN^+) cells (<40). Abbreviations: FFPE = formalin-fixed paraffin-embedded, RECAP = REcombination CAPacity, HRP = homologous recombination proficient, HRI = homologous recombination intermediate, HRD = homologous recombination deficient.

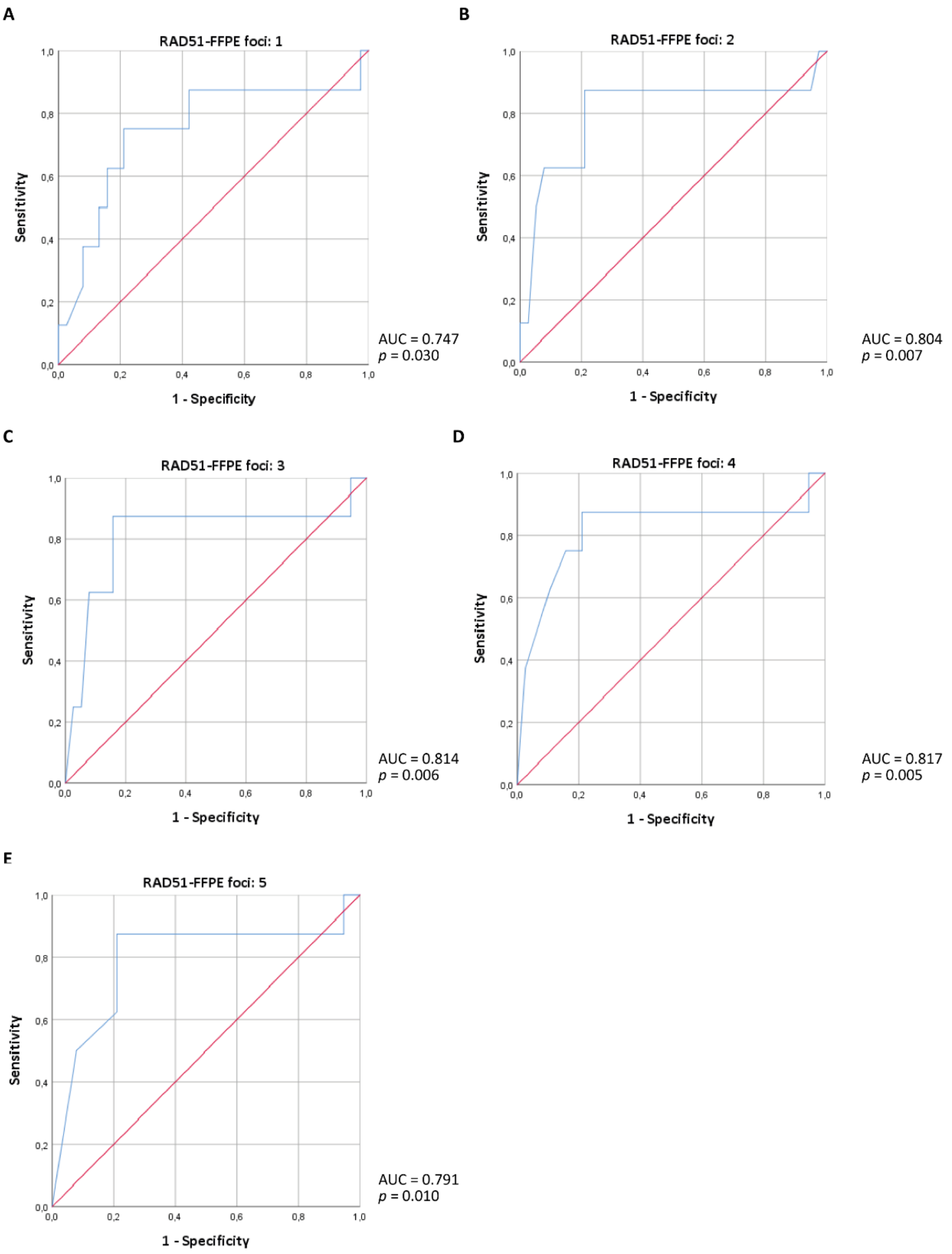

Suppl. Fig. 4 ROC curves for RAD51-FFPE scores with a RAD51 foci cut-off ranging from 1 till 5. HR classification based on RECAP scores was used as the gold standard. Tumors with RECAP scores ≤20% were considered HRD and tumors with RECAP scores of >50% were considered HRP. RECAP-HRI samples were excluded from analysis. A) ROC curve for RAD51-FFPE scores with a cut-off of 1 foci. AUC = 0.747, p = 0.030 and 95% CI (0.528-0.966). Highest sensitivity with most optimal specificity was reached at an HRD threshold of 32%, with a sensitivity of 88% and a specificity of 58%. B) ROC curve for RAD51-FFPE scores with a cut-off of 2 foci. AUC = 0.804, p = 0.007 and 95% CI (0.589-1.000). Highest sensitivity with most optimal specificity was reached at an HRD threshold of 15%, with a sensitivity of 88% and a specificity of 79%. C) ROC curve for RAD51-FFPE scores with a cut-off of 3 foci. AUC = 0.814, p = 0.006 and 95% CI (0.602-1.000). Highest sensitivity with most optimal specificity was reached at an HRD threshold of 7%, with a sensitivity of 88% and a specificity of 84%. D) ROC curve for RAD51-FFPE scores with a cut-off of 4 foci. AUC = 0.817, p = 0.005 and 95% CI (0.605-1.000). Highest sensitivity with most optimal specificity was reached at an HRD threshold of 5%, with a sensitivity of 88% and a specificity of 79%. E) ROC curve for RAD51-FFPE scores with a cut-off of 5 foci. AUC = 0.791, p = 0.010 and 95% CI (0.580-1.000). Highest sensitivity with most optimal specificity was reached at an HRD threshold of 4%, with a sensitivity of 88% and a specificity of 79%. Abbreviations: FFPE = formalin-fixed paraffin-embedded, AUC = area under curve, ROC = Receiver Operating Characteristic.

Supplementary Tables

Suppl. Table 1 Comparison of clinicopathologic characteristics between included and excluded RAD51-FFPE BC samples. Differences between the included and excluded groups were statistically tested with a *t*-test for the age at diagnosis and Fisher's Exact test for the other characteristics.

	**Included* (*n* = 63) *n* (%)**	**Excluded * (*n* = 11) *n* (%)**	***p*-value**
**Age at diagnosis** (Years ±SEM)	63.9 (1.72)	61.7 (4.38)	$p = 0.622$
**Tumor**			
Primary	53 (88)	11 (100)	$p = 0.341^{a}$
Recurrent	10 (12)		
**Histologic subtype**			$p = 0.427^{b}$
No special type (NST)	37 (70)	9 (82)	
Lobular	13 (25)	1 (9)	
Other			
Papillary	1 (2)	1 (9)	
Apocrine	1 (2)		
Cribriform	1 (2)		
**Tumor grade**			$p = 0.741^{c}$
1	5 (8)	3 (27)	
2	23 (37)	4 (36)	
3	25 (40)	4 (36)	
N/A	10 (16)		
**Hormone receptor status**			$p = 0.437^{d}$
ER+/PR+/Her2Neu-	34 (54)	6 (55)	
ER+/PR+/Her2Neu+	4 (6)		
ER+/PR-/Her2Neu-	6 (10)	2 (18)	
ER+/PR-/Her2Neu+	3 (5)	1 (9)	
ER-/PR-/Her2Neu+	1 (2)	1 (9)	
TNBC	15 (24)	1 (9)	
****NACT**			$p = 1.000^{a}$
Yes	2 (3)		
No	61 (97)	11 (100)	

Abbreviations: N/A = not applicable, HRD = homologous recombination deficient, HRP = homologous recombination proficient, NACT = neoadjuvant chemotherapy. *Due to rounding corrections, the total percentage is not always 100%. **Tumor samples that were obtained after NACT treatment. a) Fisher's exact. b) Fisher's exact, 'NST' vs 'Lobular'. c) Fisher's exact, 'grade 1-2' vs 'grade 3'. d) Fisher's exact, 'TNBC' vs 'other'.

Suppl. Table 2 Overview tumor characteristics informative study cohort (*n* = 63).

Code	RAD51-FFPE score	RECAP score	Tumor grade (NA for recurrent disease samples)	TNBC Yes/No	ER	PR	HER2 Neu	PV in HR gene(s) (LOH wildtype allele yes/no)	*TP53* PV (LOH *wt* allele yes/no)	*BRCA1* promotor methylation
BC-01[#]	14%	7%	2	No	+	+	-	*BRCA2* (yes)	No	No
BC-02	22%	94%	2	Yes	-	-	-	No	Yes (yes)	No
BC-03	32%	68%	2	No	+	+	-	No	No	No
BC-04	40%	61%	3	No	+	+	-	No	No	Unknown
BC-05	21%	88%	NA	Yes	-	-	-	No	Yes (yes)	Failed****
BC-06	52%	Failed QC	2	No	+	-	-	No	No	No
BC-07	37%	Failed QC	2	No	+	-	-	No**	Unknown	No
BC-08	50%	37%	NA	Yes	-	-	-	No	Yes (yes)	Yes
BC-09	27%	81%	3	No	+	-	+	No	No	No
BC-10	33%	100%	3	No	+	+	-	No	No	Failed****
BC-11	23%	98%	1	No	+	+	-	Unknown***	No	No
BC-12	50%	74%	NA	No	+	+	+	Unknown***	Unknown	Unknown
BC-13	3%	13%	2	No	+	+	-	No	Yes (yes)	No
BC-14	33%	88%	2	No	+	+	-	Unknown***	No	No
BC-15	33%	Failed QC	2	No	+	+	-	No	No	No
BC-16	68%	87%	NA	No	-	-	+	Unknown***	Unknown	Unknown
BC-17	3%	3%	3	No	+	+	-	No	No	No
BC-18	3%	95%	2	No	+	+	-	No	No	No
BC-19	20%	87%	3	No	+	+	-	No	No	No
BC-20	13%	92%	2	No	+	+	-	No	No	No
BC-21	68%	0%	NA	Yes	-	-	-	No	Yes (yes)	No
BC-22	0%	17%	3	Yes	-	-	-	No	Yes (yes)	No
BC-23	56%	71%	1	No	+	+	-	No	No	No

Suppl. Table 2 continued

Code	RAD51-FFPE score	RECAP score	Tumor grade	TNBC Yes/No	ER	PR	HER2 Neu	PV in HR gene(s) (LOH wildtype allele yes/no)	*TP53* PV (LOH *wt* allele yes/unknown)	*BRCA1* promotor methylation
BC-24	0%	30%	3	Yes	-	-	-	No	Yes (yes)	No
BC-25	3%	14%	3	Yes	-	-	-	No	Yes (yes)	No
BC-26*	37%	98%	2	No	+	-	-	*ATM* (no)	No	No
BC-27	15%	57%	2	No	+	+	-	No	Yes (unknown)	Unknown ***
BC-28	40%	99%	2	No	+	+	-	No**	Unknown***	Unknown ***
BC-29	16%	56%	3	No	+	-	+	No**	No	Unknown ***
BC-30	37%	89%	3	No	+	+	+	No**	No	No
BC-31	6%	Failed QC	1	No	+	+	-	No	No	No
BC-32	5%	84%	2	No	+	+	-	No	No	No
BC-33*	22%	56%	NA	Yes	-	-	-	No	Yes (yes)	No
BC-34	32%	92%	2	No	+	+	-	No**	No	No
BC-35	1%	87%	3	Yes	-	-	-	No	Yes (yes)	No
BC-36	10%	54%	2	Yes	-	-	-	*CHEK2* (no)	Yes (yes)	No
BC-37	74%	Failed QC	NA	No	+	+	-	No	No	Unknown ***
BC-38	29%	96%	2	No	+	+	-	No**	No	No
BC-39	60%	98%	3	No	+	+	-	Unknown***	Unknown***	No
BC-40	22%	95%	1	No	+	+	-	No**	No	No
BC-41	53%	94%	NA	No	+	+	-	No**	Unknown***	No
BC-42	4%	19%	3	Yes	-	-	-	No	Yes (yes)	No
BC-43	32%	100%	2	No	+	-	-	Unknown***	No	Unknown ***

Suppl. Table 2 continued

Code	RAD51-FFPE score	RECAP score	Tumor grade	TNBC Yes/No	ER	PR	HER2 Neu	PV in HR gene(s) (LOH wildtype allele yes/no)	*TP53* PV (LOH *wt* allele yes/no)	*BRCA1* promotor methylation
BC-44	8%	Failed QC	3	Yes	-	-	-	*BRCA1* (yes)	Yes (yes)	No
BC-45	14%	20%	3	No	+	+	+	*BRCA1* and *BRCA2* (yes)	No	No
BC-46	12%	Failed QC	2	No	+	+	-	No	No	No
BC-47	48%	88%	3	No	+	+	-	No	No	No
BC-48	36%	95%	3	No	+	+	-	No	No	No
BC-49	35%	86%	2	No	+	+	-	No	No	No
BC-50	10%	83%	3	No	+	+	-	No	No	Yes
BC-51	51%	76%	3	No	+	-	+	No	No	No
BC-52	74%	77%	NA	Yes	-	-	-	Unknown***	No	Unknown ***
BC-53	39%	Failed QC	2	No	+	+	-	*CHEK2* (yes)	No	No
BC-54	7%	71%	3	No	+	+	-	No	No	No
BC-55	4%	86%	3	No	+	-	-	No	Yes (yes)	No
BC-56	0%	Failed QC	3	Yes	-	-	-	No	Yes (yes)	No
BC-57	21%	55%	NA	No	+	+	-	Unknown***	No	Failed****
BC-58	59%	Failed QC	2	No	+	+	-	No	No	No
BC-59	70%	Failed QC	1	No	+	+	+	No	No	No
BC-60	4%	46%	3	Yes	-	-	-	No	Yes (yes)	No
BC-61	52%	Failed QC	2	No	+	+	-	No	No	No
BC-62	0%	Failed QC	3	No	+	-	-	No	No	No
BC-63	72%	Failed QC	3	No	+	-	-	No	No	No

Abbreviations: FFPE = formalin-fixed paraffin-embedded, RECAP = REcombination CAPacity, TNBC = triple negative breast cancer, PV = pathogenic variant, QC = quality control, NA = not applicable, NGS = next-generation sequencing. # This sample was obtained from a man. *Tumor samples that were obtained after neoadjuvant chemotherapy (NACT) treatment. **Tumor samples were only sequenced for *BRCA1* and *BRCA2*. ***Tumor samples were not sequenced. ****NGS performed, but failed.

Suppl. Table 3 Overview genetic variants identified in our BC cohort.

Code	RAD51-FFPE HR status	RECAP HR status	Gene	Variant	Class*	VAF	LOH yes/no	Reported in literature
BC-01	HRD	HRD	*BRCA2*	NM_000059.3:c.9154C>T, p. (Arg3052Trp)	5	0.75	Yes	
BC-02	HRP	HRP	*TP53*	NM_000546.5:c.659A>G, p.(Tyr220Cys)	5	0.69	Yes (1 SNP)	
			PIK3CA	NM_006218.4:c.1624G>A, p.(Glu542Lys)	5	0.65	Yes	
BC-03	HRP	HRP	*BARD1*	NM_000465.4:c.1694G>A, p.(Arg565His)	3	0.35	No	
BC-05	HRP	HRP	*TP53*	NM_000546.5:c.785G>T, p.(Gly262Val)	4	0.71	Yes (1 SNP)	
			PIK3CA	NM_006218.4:c.1035T>A, p.(Asn345Lys)	4	0.49	No	
BC-08	HRD	HRI	*TP53*	NM_000546.5:c.328delC, p. (Arg110ValfsTer13)	4	0.97	Yes	
BC-09	HRP	HRP	*PALB2*	NM_024675.4:c.1099G>A, p.(Glu367Lys)	3	0.48	No	
BC-13	HRD	HRD	*TP53*	NM_000546.5:c.536A>G, p.(His179Arg)	5	0.80	Yes	
				NM_001126114.2:c.1025A>C, p.(Ter342Ser)	3	0.88	Yes	
BC-15	HRP	N/A	*PIK3CA*	NM_006218.4:c.1624G>A, p.(Glu542Lys)	5	0.37	Unknown	
BC-20	HRD	HRP	*PIK3CA*	NM_006218.4:c.1624G>A, p.(Glu542Lys)	5	0.40	Unknown	
BC-21	HRP	HRD	*TP53*	NM_000546.5:c.580_582delCTT, p.(Leu194del)	4	0.85	Yes	
BC-22	HRD	HRD	*TP53*	NM_000546.5:c.916C>T, p.(Arg306*)	5	0.89	Yes	
BC-23	HRP	HRP	*PIK3CA*	NM_006218.4:c.1633G>A, p.(Glu545Lys)	5	0.28	No	
BC-24	HRD	HRI	*TP53*	NM_000546.5:c.517G>C, p.(Val173Leu)	5	0.35	Yes	
			RAD54L	NM_003579.4:c.777G>T, p.(Met259Ile)	3	0.09	No	
			RAD51B	NM_133509.4:c.854-3C>T	3	0.11	No	
			ATM	NM_000051.3:c.2867G>A, p.(Gly956Glu)	3	0.10	Yes	
BC-25	HRD	HRD	*TP53*	NM_000546.5:c.581T>G, p.(Leu194Arg)	4	0.85	Yes	

Suppl. Table 3 continued

Code	RAD51-FFPE HR status	RECAP HR status	Gene	Variant	Class*	VAF	LOH yes/no	Reported in literature
BC-26	HRP	HRP	*BRCA2*	NM_000059.3:c.1265A>C, p.(Asn422Thr)	3	0.14	Yes	
			CHEK2	NM_007194.4:c.480A>G, p.Ile160Met	3	0.28	No	
			ATM	NM_000051.3:c.6733G>T, p.(Glu2245Ter)	4	0.12	No	
				NM_000051.3:c.8213T>G, p.(Leu2738Ter)	5	0.11	No	
BC-27	HRD	HRP	*PIK3CA*	NM_006218.4:c.3140A>G, p.(His1047Arg)	5	0.04	Yes	
			TP53	NM_000546.5:c.814G>A, p.(Val272Met)	4	0.01	Unknown	
BC-28	HRP	HRP	*BRCA2*	NM_000059.3:c.2240A>G, p.(Glu747Gly)	3	0.63	Yes	Functional, Ikegami *et al,* 2020 [42]
BC-30	HRP	HRP	*BRCA2*	NM_000059.3:c.10123A>C, p.(Ser3375Arg)	3	0.48	No	
BC-32	HRD	HRP	*TP53*	NM_001126114.2:c.1025A>C, p.(Ter342Ser)	3	0.66	Yes	
BC-33	HRP	HRP	*TP53*	NM_000546.5:c.722C>T, p.(Ser241Phe)	5	0.90	Yes	
			PIK3CA	NM_006218.4:c.241G>A, p.(Glu81Lys)	4	0.19	No	
			ATM	NM_000051.3:c.115A>G, p.(Thr39Ala)	3	0.50	No	
BC-35	HRD	HRP	*TP53*	NM_000546.5:c.578A>G, p.(His193Arg)	4	0.81	Yes	
BC-36	HRD	HRP	*CHEK2*	NM_007194.4:c.1100delC, p.(Thr367Metfs*15)	5	0.46	Unknown	
			TP53	NM_000546.5:c.541C>T, p.(Arg181Cys)	5	0.41	Yes	
			PIK3CA	NM_006218.4:c.3140A>G, p.(His1047Arg)	5	0.32	No	
BC-37	HRP	N/A	*RAD51B*	NM_133509.4:c.315+2T>A, p.(?)	3	0.43	No	
				NM_133509.4:c.997T>C, p.(Phe333Leu)	3	0.50	No	
BC-42	HRD	HRD	*TP53*	NM_000546.5:c.785G>T, p.(Gly262Val)	4	0.53	Yes	
			BRIP1	NM_032043.3:c.2768T>G, p.(Leu923Arg)	3	0.75	Yes	
BC-44	HRD	N/A	*TP53*	NM_000546.5:c.637C>T, p.(Arg213*)	5	0.67	Yes	
			BRCA1	NM_007294.4:c.5137delG, p.(Val1713Ter)	4	0.23	Yes	
BC-45	HRD	HRD	*BRCA1*	NM_007294.4:c.2359dupG, p.(Glu787GlyfsTer3)	4	0.7	Yes	
			BRCA2	NM_000059.3:c.3865_3868delAAAT,p.(Lys1289AlafsTer3)	4	0.85	Yes	

Suppl. Table 3 continued

Code	RAD51-FFPE HR status	RECAP HR status	Gene	Variant	Class*	VAF	LOH yes/no	Reported in literature
BC-47	HRP	HRP	*PIK3CA*	NM_006218.4:c.1633G>A, p.(Glu545Lys)	5	0.24	Unknown	
			BRCA2	NM_000059.3:c.5282G>C, p.(Gly1761Ala)	3	0.11	Unknown	
			RAD51D	NM_002878.3:c.515C>T, p.(Ala172Val)	3	0.21	Unknown	
BC-49	HRP	HRP	*PIK3CA*	NM_006218.4:c.3140A>G, p.(His1047Arg)	5	0.28	Unknown	
BC-51	HRP	HRP	*PIK3CA*	NM_006218.4:c.1637A>G, p.(Gln546Arg)	5	0.34	Unknown	
			RAD54L	NM_003579.4:c.1526G>A, p.(Arg509Gln)	3	0.58	Unknown	
BC-53	HRP	N/A	*CHEK2*	NM_007194.4:c.1100delC, p.(Thr367Metfs*15)	5	0.79	Yes (1 SNP)	
			PIK3CA	NM_006218.4:c.1633G>A, p.(Glu545Lys)	5	0.36	No	
			BRCA2	NM_000059.3:c.4048C>T, p.(His1350Tyr)	3	0.30	No	
BC-55	HRD	HRP	*PIK3CA*	NM_006218.4:c.3140A>T, p.(His1047Leu)	5	0.37	No	
				NM_006218.4:c.1034A>T, p.(Asn345Ile)	4	0.20	No	
			TP53	NM_000546.5:c.660T>G, p.(Tyr220Ter)	4	0.52	Yes	
BC-56	HRD	N/A	*TP53*	NM_000546.5:c.524G>A, p.(Arg175His)	5	0.75	Yes	
BC-58	HRP	N/A	*PIK3CA*	NM_006218.4:c.1624G>A, p.(Glu542Lys)	5	0.51	No	
			BRCA2	NM_000059.3:c.4828G>A, p.(Val1610Met)	3	0.35	No	
			CHEK2	NM_007194.4:c.556A>C, p.(Asn186His)	3	0.56	Unknown	Functional, Delimitsou *et al*, 2019 and Boonen *et al*, 2022[40, 46]
BC-60	HRD	HRI	*TP53*	NM_000546.5:c.377A>G, p.(Tyr126Cys)	5	0.51	Yes	
			PPP2R2A	NM_002717.4:c.547dupA, p.(Ile183AsnfsTer4)	4	0.68	Yes	
			BRCA2	NM_000059.3:c.-40+5G>C, p.(?)	3	0.25	Yes	
BC-61	HRP	N/A	*PIK3CA*	NM_006218.4:c.1624G>A, p.(Glu542Lys)	5	0.24	Unknown	
BC-63	HRP	N/A	*ERBB2*	NM_004448.3:c.2329G>T, p.(Val777Leu)	4	0.41	Unknown	

Abbreviations: BC = breast cancer, VAF = Variant Allele Frequency, LOH = loss of heterozygosity, NA = not applicable. *Pathogenicity class using the 5-tier pathogenicity classification according to Plon *et al*, 2008:1 = benign, class 2 = likely benign, class 3 = variant of unknown significance (VUS), class 4 = likely pathogenic, and class 5 = pathogenic [37].

Suppl. Table 4 HR classification by the RECAP and RAD51-FFPE tests.

	RECAP-HRD	RECAP-HRP
**RAD51-FFPE HRD**	7	9
**RAD51-FFPE HRP**	1	29

Abbreviations: FFPE = formalin-fixed paraffin-embedded, RECAP = REcombination CAPacity, HRD = homologous recombination deficient, HRP = homologous recombination proficient.

Suppl. Table 5 Sensitivity and specificity analysis for the RAD51-FFPE test with the RECAP test serving as gold standard. Commonly used HRD threshold en RAD51-FFPE foci cut-off parameters were applied to determine the sensitivity and specificity of the RAD51-FFPE test. A 5% HRD threshold with a RAD51 foci cut-off 4 and a 15% HRD threshold with a RAD51 foci cut-off of two led to the highest sensitivity and specificity for the identification of RECAP-HRD.

**HRD threshold**	**Foci number**	**RECAP-HRD** ***n*** **= 8**	**RECAP-HRP** ***n*** **= 38**	**All** ***n*** **= 46**
**5%**	**1**	25	92	80
	**2**	63	89	85
	**3**	75	84	83
	**4**	88	76	78
	**≥5**	88	68	72
**10%**	**1**	50	87	80
	**2**	63	82	78
	**3**	88	71	74
	**4**	88	61	65
	**≥5**	88	45	52
**15%**	**1**	63	82	78
	**2**	88	76	78
	**3**	88	63	67
	**4**	88	37	46
	**≥5**	88	29	39
**20%**	**1**	75	76	76
	**2**	88	71	74
	**3**	88	39	48
	**4**	88	26	37
	**≥5**	88	24	35

Abbreviations: RECAP Recombination CAPacity test, HRD = homologous recombination deficient, HRP = homologous recombination proficient.

Suppl. Table 6 Clinicopathologic characteristics stratified for HR status as determined by the RECAP test.

	*HRD (*n*= 8) *n* (%)	*HRP (*n*= 38) *n* (%)	*P*-value
**Age at diagnosis** (Years ± SEM)	68.3 (±5.52)	61.9 (±2.16)	p = 0.243[a]
**Tumor**			p = 1.000[b]
Primary	7 (88)	31 (82)	
Recurrent	1 (13)	7 (18)	
**Histologic subtype (primary)**			p = 1.000[c]
No special type (NST)	6 (86)	23 (74)	
Lobular	1 (14)	6 (19)	
Other			
Papillary		1 (3)	
Apocrine		1 (3)	
Cribriform			
**Tumor grade**			p = 0.405[d]
1	-	3 (8)	
2	2 (25)	14 (37)	
3	5 (63)	14 (37)	
NA	1 (13)	7 (18)	
**Hormone receptor status**			p = 0.055[e]
TNBC	4 (50)	6 (16)	
Other			
ER+/PR+/Her2Neu-	3 (38)	23 (61)	
ER+/PR+/Her2Neu+	1 (13)	2 (5)	
ER+/PR-/Her2Neu-	-	3 (8)	
ER+/PR-/Her2Neu+	-	3 (8)	
ER-/PR-/Her2Neu+	-	1 (3)	
***TP53* PV**			p = 0.206[b]
Yes	5 (63)	7 (30)	
No	3 (38)	16 (70)	
***BRCA1/2* PV**			**p = 0.040[b]**
Yes	2 (25)	-	
No	6 (75)	30 (100)	
***BRCA1* promotor hypermethylation**			p = 1.000[b]
Yes	-	1 (3)	
No	8 (100)	29 (97)	

Significant *p* values are indicated in bold. Abbreviations: NA = not applicable, RECAP = REcombination CAPacity, HRD = homologous recombination deficient, HRP = homologous recombination proficient, PV = pathogenic variant. *Due to rounding corrections, the total percentage is not always exactly 100%. a) Student's *t*-test. b) Fisher's exact c) Fisher's exact, 'NST' vs 'lobular'. d) Fisher's exact, 'grade 1-2' vs 'grade 3'. e) Fisher's exact, 'TNBC' vs 'other'.
